# Adsorption Characteristics of Bixin on Acid- and Alkali-Treated Kaolinite in Aprotic Solvents

**DOI:** 10.1155/2018/3805654

**Published:** 2018-01-18

**Authors:** Winda Rahmalia, Jean-François Fabre, Thamrin Usman, Zéphirin Mouloungui

**Affiliations:** ^1^Université de Toulouse, INP-ENSIACET, Laboratoire de Chimie Agro-industrielle (LCA), 4 Allée Monso, 31030 Toulouse, France; ^2^Department of Chemistry, Mathematics and Natural Science, Tanjungpura University, Jl. Ahmad Yani, Pontianak 78124, West Kalimantan, Indonesia; ^3^INRA, UMR 1010 CAI, 31030 Toulouse, France

## Abstract

The adsorption of bixin in aprotic solvents onto acid- and alkali-treated kaolinite was investigated. Kaolinite was treated three times, for 6 h each, with 8 M HCl or 5 M KOH. The adsorbents were characterized by XRD, FT-IR, EDS, and BET-N_2_. The effects of contact time and dye concentration on adsorption capacity and kinetics, electronic transition of bixin before and after adsorption, and also mechanism of bixin-kaolinite adsorption were investigated. Dye adsorption followed pseudo-second order kinetics and was faster in acetone than in dimethyl carbonate. The best adsorption results were obtained for KOH-treated kaolinite. In both of the solvents, the adsorption isotherm followed the Langmuir model and adsorption capacity was higher in dimethyl carbonate (*q*_*m*_ = 0.43 mg/g) than in acetone (0.29 mg/g). The adsorption capacity and kinetics of KOH-treated kaolinite (*q*_*m*_ = 0.43 mg/g, *k*_2_ = 3.27 g/mg·min) were better than those of HCl-treated kaolinite (*q*_*m*_ = 0.21 mg/g, *k*_2_ = 0.25 g/mg·min) and natural kaolinite (*q*_*m*_ = 0.18 mg/g, *k*_2_ = 0.32 g/mg·min). There are shift in the band position of maximum intensity of bixin after adsorption on this adsorbent. Adsorption in this system seemed to be based essentially on chemisorption due to the electrostatic interaction of bixin with the strong basic and reducing sites of kaolinite.

## 1. Introduction

Bixin (methyl hydrogen 9′-*cis*-6,6′-diapocarotene-6,6′-dioate, C_25_H_30_O_4_) is a carotenoid dye extracted from the seeds of the tropical shrub annatto (*Bixa orellana* L.) [1]. It is widely used in industry, cosmetics, and pharmaceutical products and as a food colouring and textile dye [2–5]. Bixin has photoactive properties and was recently explored as a sensitizing dye in solar cells [6–8] and for photodynamic therapy [9]. The potential uses of bixin in these applications are based on the conjugated double bond which can absorb energy in the visible region (400–500 nm), yielding colours in the yellow, orange, and red range. However, as other carotenoids, this double bond renders bixin unstable to light, temperature, and oxygen exposure [10, 11].

The poor heat and light stability of carotenoids in vitro is problematic when trying to construct photofunctional materials, such as photosensitized semiconductors and nonlinear optical materials [12]. There have been reports of efforts to increase the stability of bixin to render it suitable for a broader range of applications, by incorporating this molecule into the surface or interlayer space of clay minerals. Kohno et al. [13] showed that annatto dye/organo-montmorillonites were more photostable than pure annatto dye because the layered structure of the montmorillonite protected the dye molecules from external oxygen. Rahmalia [14] reported the immobilization of bixin on natural kaolinite. The resulting product had slower degradation kinetics in acetone than pure bixin. Furthermore, solar cells sensitized with bixin immobilized on acid-activated kaolinite [7] had a higher energy conversion efficiency than cells sensitized with pure bixin [6].

The incorporation of various organic compounds into clays and clay minerals has been reported, due to the surface properties of these minerals, such as their adsorption capacities, surface charges, their large surface area, charge density, types of exchangeable cations, hydroxyl groups on the edges, silanol groups of crystalline defects or broken surfaces, and Lewis and Brönsted acidity [15–17]. Kaolinite (Al_2_Si_2_O_5_(OH)_4_) is a relatively inexpensive clay mineral that is highly effective as a carrier material. This behaviour is governed by the extent and nature of the external surface, which can be modified by appropriate treatment techniques [18–20]. Activation with acid or alkali has been widely studied as a chemical treatment for improving the surface characteristics of natural kaolinite in terms of its interactions with adsorbate [21–23]. Kaolinite is also hydrophobic and could therefore easily adsorb hydrophobic organic molecules, such as bixin [24–27]. The use of an activation process without heating for the ultimate enhancement of energy economics for bixin was investigated.

Clay minerals have been used as solid matrices to enhance the stability of bixin, but no systematic study has described the mechanism of bixin adsorption onto kaolinite. Rapid and efficient dye adsorption is important for industrial applications. The isotherm and adsorption kinetics of bixin were therefore determined on acid- and alkali-treated kaolinite. Untreated kaolinite was also tested for the purpose of comparison. Acetone and dimethyl carbonate were used as solvents because bixin was highly soluble in both these aprotic solvents. We previously reported that the transition energy and molar attenuation coefficient of bixin in dimethyl carbonate were similar to those in acetone [28]. Dimethyl carbonate would therefore be an appropriate alternative to volatile organic solvents like the nonpolar aprotic solvent acetone. Dimethyl carbonate is widely used as dialkyl carbonate with many applications in novel green chemistry [29]. It is a valuable chemical for industrial chemical engineering, due also to its low toxicity [30]. This study identified appropriate ketone and carbonate solvents for use in applications of bixin. Such applications, including the synthesis of a new sensitizing kaolinite-bixin dye, have become important in several fields.

## 2. Materials and Methods

### 2.1. Materials

Bixin crystals containing 88.11% *cis*-bixin and 11.75% di-*cis*-bixin and an unknown compound (0.14%) were obtained by the extraction and purification processes described by Rahmalia et al. [31]. Kaolinite (Al_2_O_7_Si_2_.2H_2_O), CAS Number 1318-74-7, was supplied by Sigma-Aldrich (Germany), together with analytical grade hydrochloric acid (HCl, 37%) and potassium hydroxide (KOH), and the HPLC grade solvents acetone (≥99.5%) and dimethyl carbonate (99%) were supplied by Sigma-Aldrich (Germany).

### 2.2. Methods

#### 2.2.1. Preparation of Acid- and Alkali-Treated Kaolinite

Activation was achieved by adding 10 g of kaolinite to 100 mL 8 M HCl or 100 mL 5 M KOH separately. The mixtures were incubated at room temperature for 6 h, with constant shaking (300 rpm). The suspension was filtered, and the residue was washed with distilled water until neutral and then dried in an oven at 103°C for 24 h. This process was repeated three times to optimize activation. The final products obtained are referred to as KA for HCl-treated kaolinite and KB for KOH-treated kaolinite. The untreated sample is referred to as KN.

#### 2.2.2. Characterization of the Adsorbent

X-ray diffraction (XRD) patterns for the samples were obtained with a Bruker X-ray diffractometer (CuK*α*, *λ* = 1.54 Å, step scan size of 0.02° and a count time of 0.5 seconds, 25°C). Samples were prepared by allowing the particles of kaolinite to settle in water to obtain 2 *μ*m particles. They were then treated with ethylene glycol and prepared as oriented mounts on a glass slide.

Fourier transform infrared spectroscopy (FT-IR) was carried out on a Shimadzu FT-IR spectrophotometer, over a spectral region of 400–4000 cm^−1^, with a resolution of 1 cm^−1^, and samples were evaluated in powder form mixed with KBr powder.

The BET surface area of the samples was determined by the multipoint N_2_ adsorption-desorption method at the temperature of liquid nitrogen (−196°C), with an ASAP 2010 (Micrometrics) instrument.

Changes in elemental composition after treatment were assessed with an Edax Ametek high-resolution energy dispersive X-ray spectroscopy (EDS) detector.

#### 2.2.3. Adsorption Experiment

Stock solutions of bixin (20 mg/L) were prepared in acetone and dimethyl carbonate separately. Solutions of the required concentration (3–18 mg/L) were prepared by diluting the stock solution. Adsorbent (0.05 g) was then added to 5 mL bixin solution (3–18 mg/L). The mixtures were incubated at room temperature (∼22°C), with shaking at 300 rpm. The samples were withdrawn after 4 h (predetermined equilibrium time), and small aliquots of the supernatant were removed and diluted to an appropriate concentration if required. The absorption spectrum was determined immediately with a Shimadzu UV-1800 UV-Vis spectrophotometer. The bixin concentration of the solutions was determined with a UV spectrophotometer calibrated at 457 nm for acetone and 456 nm for dimethyl carbonate [28]. For the contact time studies, the residual concentration of the 5 mL bixin solution (±10 mg/L) with kaolinite (0.1 g) was determined at various time points, from 5 to 360 min. The experiments were carried out in triplicate.

## 3. Results and Discussion

### 3.1. Characterization of Adsorbents

The XRD patterns of KN (Supporting Information Figure S1) showed two intense diffraction reflections at 2*θ* values of 12.3 and 24.9°, less intense reflections at 2*θ* values of 23.2 and 26.6°, and a hump at 2*θ* values of 19.8–21.5°, associated with kaolinite (PDF 00-058-2001). The diffraction reflections of orthoclase were found at 2*θ* values of 15.4, 21.0, 25.7, 27.5, and 30.1° (PDF 00-022-1212), whereas the diffraction reflections of muscovite were found at a 2*θ* value of 18.0° (PDF 00-058-2036). After treatment with KOH, the reflection width and intensity of kaolinite decreased at 2*θ* values of 12.3 and 24.9°. This decrease was attributed to a minor structural disorder resulting from alkali treatment, which affects the crystalline nature of the clay [32].

**Figure 1:**
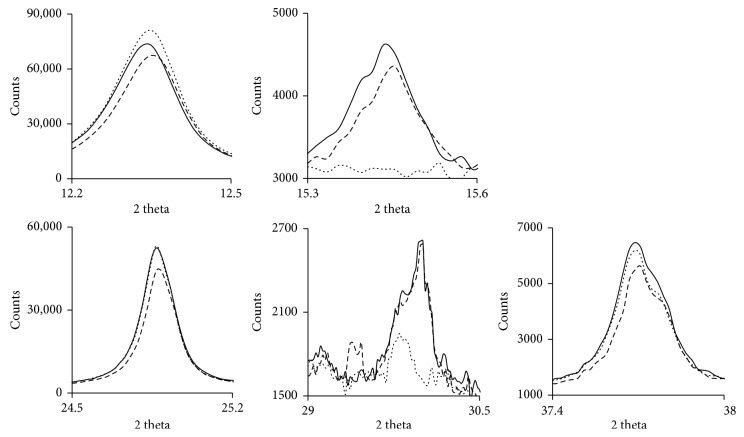
X-ray diffraction (XRD) patterns of KN (—), KA (....), and KB (----).

A diffractogram for KA showed no significant difference with respect to KN, but 2*θ* values of 15.4 and 30.1° were unobservable, and the reflection increased at a 2*θ* of 12.3° (Figure 1). This finding may reflect the greater resistance to acid attack of the structure of kaolinite than that of orthoclase. The resistance of clay minerals to acid attack depends strongly on their crystallinity, with more regular crystals associated with greater resistance to acid attack [33]. It may also be due to the elimination of mineral impurities by acid leaching. The higher peak intensity may reflect the presence of larger crystallites or a decrease in mean lattice strain [32, 34].

**Figure 2:**
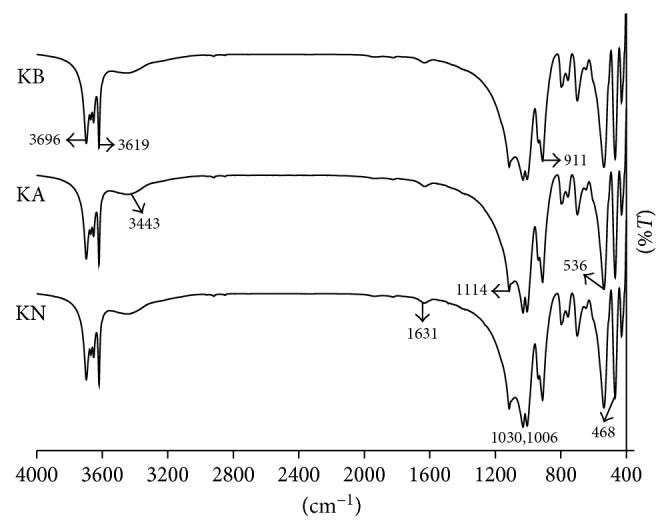
FT-IR spectra of KN, KA, and KB.

The FT-IR spectra of KN, KA, and KB (Figure 2) revealed bands at 3696, 3669, 3652, and 3619 cm^−1^ corresponding to the stretching of inner-surface hydroxyl groups, at 3443 cm^−1^ corresponding to stretching of the hydroxyl group of water, 1631 cm^−1^ corresponding to the O–H deformation of water, 1114 cm^−1^ corresponding to Si–O stretching (longitudinal mode), 1030 and 1006 cm^−1^ corresponding to in-plane Si–O stretching, 937 cm^−1^ corresponding to the OH deformation of inner-surface hydroxyl groups, 911 cm^−1^ corresponding to Al–OH deformation, 794 cm^−1^ corresponding to Si–O vibration, 755 and 696 cm^−1^ corresponding to Si–O perpendicular vibrations, 536 cm^−1^ corresponding to Al–O–Si deformation, 468 cm^−1^ corresponding to Si–O–Si deformation, and 428 cm^−1^ corresponding to Si–O deformation [35, 36]. The FT-IR spectra patterns of KN, KA, and KB showed no significant differences between kaolinite before and after treatment, indicating an absence of significant change in the kaolinite samples. Infrared absorption spectra, which displayed a fairly sharp absorption band at wave numbers around 911 cm^−1^ for both samples, showed an absence of change in the composition of the octahedral Al atoms following treatment with acid and alkali in the experimental conditions used.

Changes in elemental composition were investigated by EDS (Table 1). The samples of kaolinite tested were rich in silicon and aluminium. Their composition in terms of impurities, such as Na, Mg, K, and Fe, depended on the type of reagent used. Si and Al contents only slightly decreased after treatment. This shows the resistance of natural kaolinite minerals to acid and alkali attack.

The results of nitrogen sorption isotherm analysis are summarised in Table 2. The surface area, pore volume, and mean diameter of the untreated kaolinite sample were 7.65 m^2^/g, 3.62 × 10^−2^ cm^3^/g, and 18.9 nm, respectively, indicating that the porosity of the original kaolinite was low. Pore volume and mean diameter increased after acid treatment, possibly due to the dissolution of metal ions present in the kaolinite and the rearrangement of its crystal structure, as a result of a reaction between the acid and the clay mineral. Treatment with 5 M alkali at room temperature has been shown to increase specific surface area but to decrease total pore volume and mean diameter. Belver et al. [21] reported an increase in specific surface area, presumably because of the disaggregation/separation of kaolinite particles. The decrease in specific surface area observed for KA may be due to an increase in crystallinity, as indicated by XRD.

### 3.2. Electronic Transition of Bixin

**Figure 3:**
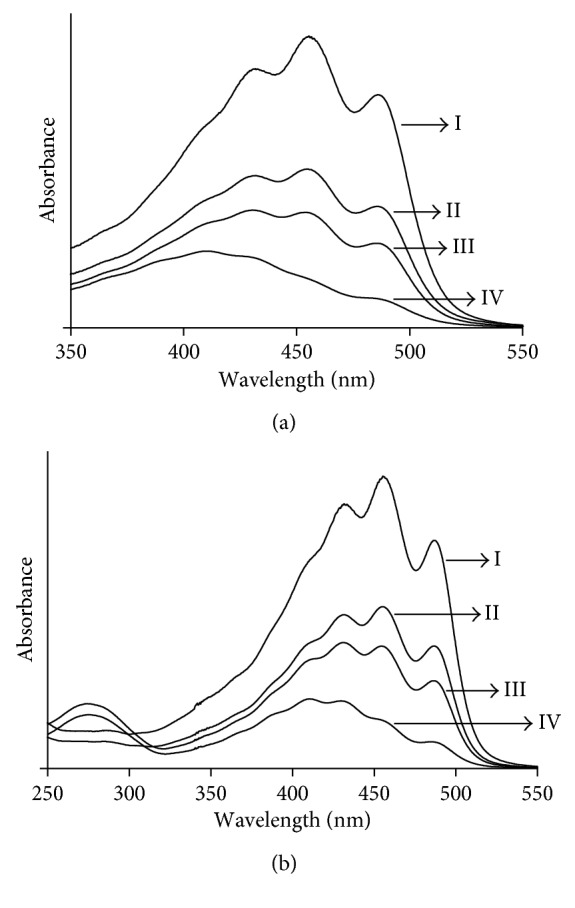
Absorption spectra of bixin in acetone (a) and dimethyl carbonate (b) before adsorption (I) and supernatant solution of bixin after adsorption on KN (III), KA (II), and KB (IV).

The absorption spectrum of the supernatant of bixin in acetone and dimethyl carbonate was in the visible region, with peaks at 457 nm and 456 nm, respectively (Figure 3), associated with the 0-1 vibration band position, consistent with the finding of Rahmalia et al. [28]. After adsorption onto KA, the maximum intensity (λmax) of the bixin spectrum was still in the 0-1 band position. However, after adsorption onto KN and KB, the maximum wavelength shifted to shorter wavelengths, associated with a band position of 0–2 for KN and of 0–3 for KB, and intensity decreased. Schoonheydt and Johnston [37] reported that the absorption maxima of dye molecules in nonpolar solvents adsorbed onto the surface of clay minerals could shift to shorter wavelengths because the solvent-molecule interaction was stronger in the ground state than in the excited state. Yariv and Cross [38] suggested that the absorption band of the adsorbed dye displayed a blue shift due to interactions between the π-electrons of the dye and the hybridised orbitals of the surface oxygen atoms, leading to a stabilisation of the π-orbitals and a destabilisation of the π^∗^-orbitals.

### 3.3. Effect of Contact Time

**Figure 4:**
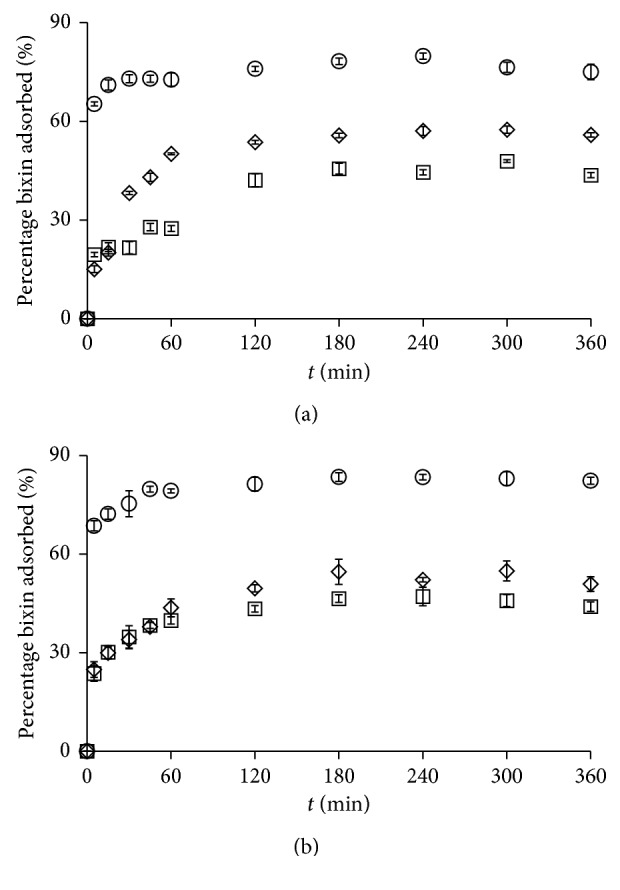
Effect of contact time on adsorption of bixin using KN (◇), KA (□), and KB (○) in acetone (a) and dimethyl carbonate (b).

The effects of contact time on the amount of bixin adsorbed onto kaolinite were investigated (Figure 4). Kaolinites adsorb bixin with different efficiencies, and bixin was rapid and strong during the initial period of contact, between 5 and 60 minutes. During this period, the tendency towards adsorption was high, and the slope of the adsorption curve was steep. This early phase of steep increase was followed by a phase of slow increase between 120 and 360 minutes. During this period, the slope of the adsorption curve gradually flattened out, and the bixin adsorption gradually decreased eventually reaching zero. This corresponded to equilibrium being reached due to the saturation of adsorption sites.

The single, smooth, and continuous nature of the curves suggested that the bixin might cover the kaolinite as a monolayer. The percentage dye adsorption was highest on KB, consistent with the BET specific surface area analysis, which indicated that adsorption was most likely to occur on the external surface of kaolinite. It took 180 minutes to reach equilibrium for bixin in acetone with KN as the adsorbent and 240 minutes for the same mixture but with KA or KB as the adsorbent. It took 240 minutes to reach equilibrium for bixin in dimethyl carbonate, with KN or KB as the adsorbent, and 300 minutes for the same mixture but with KA as the adsorbent. This phenomenon is influenced by the surface properties of the adsorbent and the chemical and physical constants of the solvents.

### 3.4. Effect of Initial Dye Concentration

**Figure 5:**
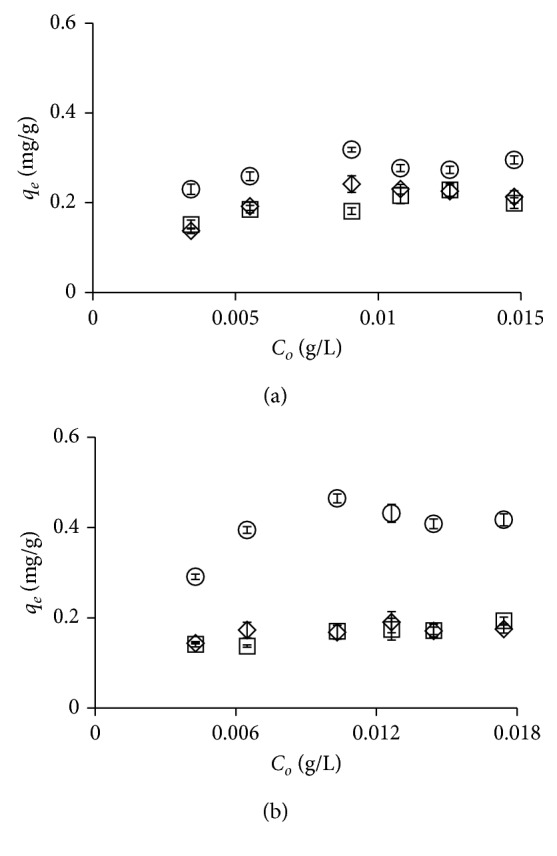
Effect of initial dye concentration on adsorption of bixin using KN (◇), KA (□), and KB (○) in acetone (a) and dimethyl carbonate (b).

The effect of initial dye concentration on equilibrium adsorption was investigated at different initial bixin concentrations. Initial bixin concentration affected the amount of bixin adsorbed at equilibrium (Figure 5). At low initial bixin concentrations, the adsorption capacity of KN, KA, and KB increased with initial bixin concentration. It therefore seems likely that an increase in adsorption with initial dye concentration leads to an increase in mass gradient between the solution and adsorbent, thereby driving the transfer of additional dye molecules from the bulk solution to the particle surface [39].

### 3.5. Adsorption Isotherm

Adsorption properties and equilibrium parameters, commonly known as adsorption isotherms, describe the interaction of the adsorbate with the adsorbents, improving understanding of the nature of the interaction. Isotherms provide information about the optimal use of adsorbents. When optimizing the design of an adsorption system, it is essential to establish the most appropriate correlation for the equilibrium curve. Several isotherm equations are available for analysis of experimental sorption equilibrium parameters. However, Langmuir and Freundlich models are the most widely used type of isotherm [15, 23–25, 33, 40]. These models were used to explain the interaction between bixin and kaolinite in this study. They are the best models for explaining adsorption trends and are based on the rationale that the adsorbents become saturated with adsorbate after sufficiently long contact times.

The Freundlich isotherm describes the nonspecific adsorption of a heterogeneous system and reversible adsorption. The linear form of the Freundlich equation (1) is expressed as follows [41]:

(1) $$\begin{array}{c} \text{log}q_{e}=\text{log}K_{F}+\frac{1}{n}\text{log}C_{e}, \end{array}$$

where 1/*n* is a combined measurement of the relative magnitude and diversity of energies associated with a particular sorption process.

In the Langmuir model, the mass of solute adsorbed per unit mass of adsorbent increases linearly with solute concentration at low surface coverage, approaching an asymptote as the adsorption sites become saturated. Equation (2) is based on three important assumptions: (1) the energy of adsorption is identical for all sites and is independent of surface coverage, (2) adsorption occurs only at localised sites, with no interaction between adjoining adsorbed molecules, and (3) the sorption maximum represents monolayer coverage. The linear form of the Langmuir equation (2) can be expressed as follows [42]:

(2) $$\begin{array}{c} \frac{C_{e}}{q_{e}}=\frac{1}{K_{L}\cdot q_{m}}+\left(\frac{1}{q_{m}}\right)C_{e}. \end{array}$$

We calculated the values of the parameters of the Freundlich and Langmuir model (Table 3). The equilibrium data were not consistent with the Freundlich equation for all adsorbents, in either of the two solvents. The poor fit of this model was demonstrated by the very low correlation coefficient (*r* ^2^ < 0.95). The values of 1/*n* < 1 indicates a nonlinear adsorption of the Freundlich model and corresponds to a Langmuir-type isotherm curve, in which marginal sorption energy decreases with increasing surface concentration. The Langmuir equation gave a better fit, with *r* ^2^ > 0.95, indicating a homogeneous active site and the coverage of the adsorbent surface with a monolayer of bixin. Based on *q*_*m*_ values, bixin adsorption to KB was more favourable than its adsorption to KA and KN.

**Figure 6:**
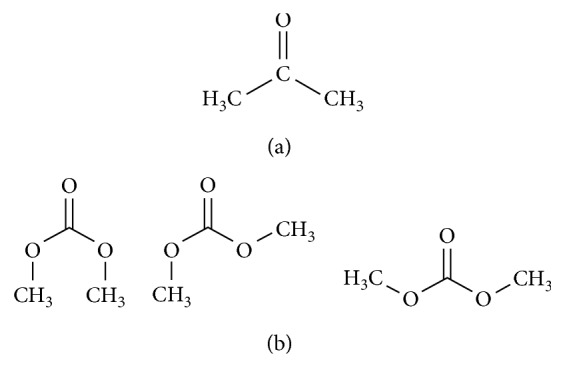
Structure conformations of acetone (a) and dimethyl carbonate (b).

The adsorption capacity of the adsorbents appeared to increase with specific surface area. The capacity of bixin to adsorb to adsorbents may reflect the extent to which the kaolinite was able to swell. The physical swelling of the kaolinite probably depended on the bulk properties of the intervening solvent molecules. In nonpolar solvents, increase in dielectric constant function (*R*(*ε*)) is associated with decreases in the volume of the kaolinite, due to lower levels of physical swelling [43]. Adsorption capacity was therefore greater when dimethyl carbonate (*R*(*ε*) = 0.412) was used as a solvent, because this solvent has lower dielectric constants than acetone (*R*(*ε*) = 0.872). The dimethyl carbonate also has several conformations (at least 3) against acetone which has only one conformation (Figure 6), causing the possibility of DMC molecules in the highly aggregated solvate. Two structure conformations of DMC (Figure 6b) are favourable between DMC and KB which oxygen of DMC interact with metal atom of Si and Al of KB [44–46].

### 3.6. Adsorption Kinetics

Lagergren's pseudo-first order and pseudo-second order models were used to investigate the dynamics of bixin adsorption onto kaolinite. The pseudo-first order model assumes that the rate of change of solute uptake over time is directly proportional to the difference in saturation concentration and the amount of solid uptake over time. In most cases, the adsorption reaction involves diffusion across a boundary (3) [47]. The adsorption process with chemisorptions controls the rate, according to the pseudo-second order model (4) [48].

(3) $$\begin{array}{c} \text{log }\left(q_{e}-q_{t}\right)=\text{log }q_{e}-k_{1}\cdot \frac{t}{\left(2.303\right)}, \end{array}$$

(4) $$\begin{array}{c} \frac{t}{q_{t}}=\frac{1}{\left(k_{2}q_{e}^{2}\right)}+\left(\frac{1}{q_{e}}\right)\cdot t. \end{array}$$

*k*_1_ and *k*_2_ were calculated from the intercept of the corresponding plots of log (*q*_*e*_–*q*_*t*_) against *t* and *t*/*q*_*t*_ against *t*. They are shown in Table 4, along with the values for the correlation coefficients, *q*_*e*1_ and *q*_*e*2_ (calc.) and *q*_*e*_ (exp.). The correlation coefficient values for the pseudo-second order rate equation were higher than those for the pseudo-first order rate equation (Table 4). The *r* ^2^ values for the plots were in the range 0.65–0.97 after application of the pseudo-first order model, but the calculated *q*_*e*1_ values obtained with this model did not give reasonable values because they were lower than the experimental *q*_*e*_ values. The *q*_*e*2_ and *q*_*e*_ values were very similar for the pseudo-second order model.

The adsorption process on all adsorbents in both solvents was found to follow the pseudo-second order kinetic model. These results suggest that chemisorption predominated in the adsorption occurring in this work [17]. The best results were obtained for KOH-treated kaolinite. Adsorption kinetics was faster with acetone (*k*_2_ = 3.27) than with dimethyl carbonate (*k*_2_ = 1.08) as the solvent. The smaller size of acetone molecules than of dimethyl carbonate molecules and the lower viscosity of acetone (0.295 cP) than of dimethyl carbonate (0.585 cP) may facilitate the diffusion of bixin into the interlayer region of kaolinite.

### 3.7. Mechanism of Bixin-Kaolinite Adsorption

**Figure 7:**
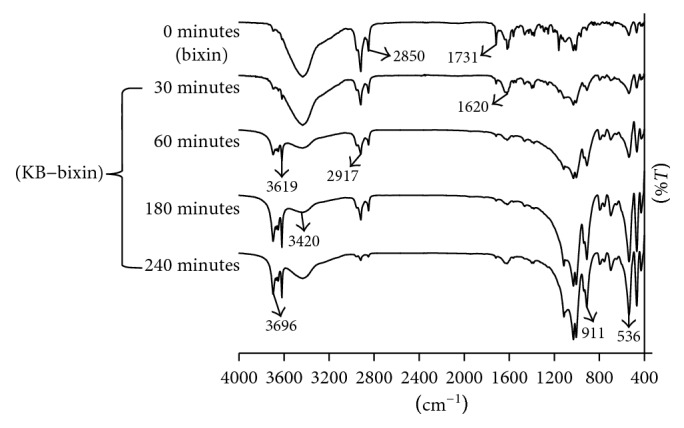
FT-IR spectra of bixin dye and bixin-KB obtained from the processes of adsorption in different times.

The FT-IR spectra of bixin dye and of bixin-KB following adsorption for different times were obtained (Figure 7). The spectrum of bixin dye may be assigned as follows: the –O–H stretching vibration at 3420 cm^−1^, the H–C–H bending vibration at 2957, 2917, and 2850 cm^−1^, the C=O ester group at 1731 cm^−1^, the O–H bending vibration at 1620 cm^−1^, the alkene C=C stretching at 1469 cm^−1^, C–H bending of the methyl groups at 1378, C=O stretching at 1220 cm^−1^, symmetric and asymmetric vibrations of the C–O–C ester group at 1180 cm^−1^, and the methylene rocking vibration of *cis*-carotenoid at 720 cm^−1^ [49].

Figure 7 shows significant frequency modifications of the absorption bands from bixin in low frequency areas between 3400 and 3700 cm^−1^ and in high frequency areas between 1600 and 1750 cm^−1^. Functional groups COOH and COOR from bixin strongly absorb at strong basic and reducing sites of KN through electrostatic interactions. The frequency of bands at 3420 cm^−1^ disappears in favour of frequency of bands at 3619 cm^−1^. The same phenomenon was observed in high frequency areas; the frequency of bands at 1731 cm^−1^ disappears in favour of frequency of bands at 1620 cm^−1^. This indicated strong interactions between two types of metal carboxylate groups, which result from a part of the interaction between the carboxylic group of the bixin and the other part of the carboxyester group of bixin, respectively, with Si and Al.

## 4. Conclusion

The adsorption characteristics of bixin onto kaolinite, especially for constructing photofunctional materials based on kaolinite-bixin organoclay, have been investigated. This adsorption is more dependent on the specific surface area of the adsorbent. The adsorption capacity of the kaolinite was considerably improved by an increase in the surface specific area. Alkali treatment (BET specific surface area = 8.16 m^2^g^−1^) was therefore more suitable than acid treatment for increasing the capacity of kaolinite to adsorb organic molecules, such as bixin. Selection of the most appropriate aprotic solvent also increased the efficiency of bixin absorption onto kaolinite. Based on UV-visible spectroscopy data, the solvent-molecule interaction was stronger in the ground state than in the excited state. The adsorption isotherm was of the Langmuir-type and was higher in acetone than in dimethyl carbonate. Dye adsorption followed pseudo-second order kinetics and was faster in dimethyl carbonate complex solvate than in acetone. Adsorption in this system appears to be mostly due to chemisorption mediated by the electrostatic interaction of bixin with the strong basic and reducing sites of kaolinite. Finally, dimethyl carbonate has potential as a good solvent with no compound organic volatile for increasing bixin adsorption onto kaolinite.

## Figures and Tables

**Table 1:** EDS analysis data.

Element	wt. %		
	KN	KA	KB
O	56.9	59.0	56.4
Na	0.42	0.37	0.33
Mg	0.15	0.06	0.37
Al	18.4	17.9	18.1
Si	23.1	21.3	22.7
K	0.79	0.55	0.93
Fe	0.22	0.34	0.33
Si/Al	1.26	1.19	1.25

**Table 2:** Nitrogen sorption isotherm data.

Sample	BET specific surface area, *a*_*s*_ BET (m^2^g^−1^)	Total pore volume (10^−2^ cm^3^g^−1^)	Mean pore diameter, *d*_*p*_ (nm)
KN	7.65	3.62	18.9
KA	7.28	3.74	20.6
KB	8.16	2.85	14.0

**Table 3:** Adsorption isotherm parameters for the adsorption of bixin on kaolinite.

Solvent	Adsorbent	Freundlich			Langmuir		
		*K*_*F*_	1/*n*	*r* ^2^	*K*_*L*_	*q*_*m*_	*r* ^2^
Acetone	KN	0.13	0.25	0.7043	1.16	0.24	0.9747
	KA	0.14	0.17	0.7107	1.22	0.22	0.9691
	KB	0.23	0.10	0.6005	4.00	0.29	0.9893
Dimethyl carbonate	KN	0.14	0.10	0.5600	2.03	0.18	0.9915
	KA	0.11	0.19	0.8362	0.48	0.21	0.9807
	KB	0.32	0.14	0.5701	5.00	0.43	0.9927

**Table 4:** Adsorption kinetics parameters for the adsorption of bixin on kaolinite.

Solvent	Adsorbent	*q*_*e*_ exp	Pseudo-first order			Pseudo-second order			
			*q*_*e*1_	*k*_1_ (10^−2^)	*r* ^2^	*q*_*e*2_	*h* (10^−2^)	*k*_2_	*r* ^2^
Acetone	KN	0.25	0.21	2.53	0.9697	0.26	2.07	0.32	0.9951
	KA	0.21	0.16	1.17	0.8969	0.21	1.07	0.25	0.9823
	KB	0.35	0.09	1.70	0.6928	0.33	35.7	3.27	0.9956
Dimethyl carbonate	KN	0.29	0.20	1.77	0.9300	0.28	2.84	0.35	0.9947
	KA	0.25	0.15	1.77	0.9549	0.24	4.05	0.68	0.9962
	KB	0.45	0.12	1.31	0.6456	0.44	20.5	1.08	0.9991

## Nomenclature and Units

*q*_*e*_: Amount of dye adsorbed per unit of adsorbent at equilibrium (mg/g)
*C*_*o*_: Initial concentration of the dye solution (mg/L)
*C*_*e*_: Concentration of the dye solution at adsorption equilibrium (mg/L)
*K*_*F*_: Freundlich adsorption isotherm constant (mg^1-1/n^L^1/n^g)
*N*: Freundlich adsorption isotherm constant
*K*_L_: Langmuir constant (L/mg)
*q*_*m*_: Maximum adsorption resulting in monolayer coverage on the adsorbent surface (mg/g)
*q*_*t*_: Amount of dye adsorbed per unit mass adsorbent at any time t (mg/g)
*k*_1_: Pseudo-first order adsorption rate constant (1/min)
*k*_2_: Pseudo-second order adsorption rate constant (g/mg·min)
*H*: Initial adsorption rate at any time approaching 0 (mg/g·min)
*R*(*ε*): Dielectric constant function.

## Supplementary Materials

Supplementary Materials Figure S1. XRD patterns of KN.
