# Supplementary figures and images for: Adsorption Characteristics of Bixin on Acid- and Alkali-Treated Kaolinite in Aprotic Solvents
Source: Bioinorg Chem Appl. 2018 Jan 18;2018:3805654. doi: 10.1155/2018/3805654 (PMC5822873; doi:10.1155/2018/3805654)

**SUPPORTING INFORMATION**


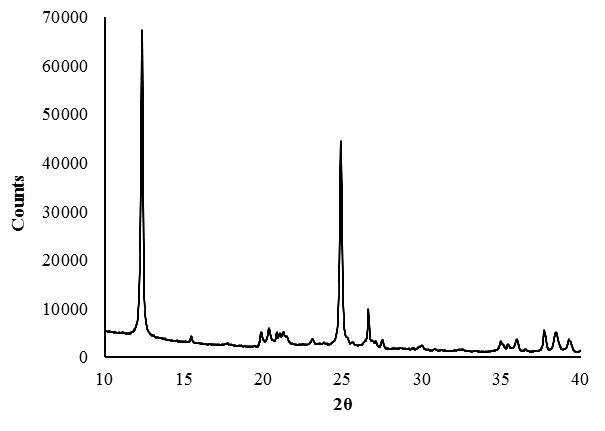


Figure S1 XRD patterns of KN

Supplement: Supplementary Materials — Figure S1. XRD patterns of KN. [file 3805654.f1.doc]
